# Tuberculosis in the South America’s homeless: a systematic review of social determinants, control strategies and health equity

**DOI:** 10.1590/1980-220X-REEUSP-2025-0222en

**Published:** 2026-05-01

**Authors:** Eliza Miranda Ramos, Carolina Braga Sisti, Sérgio Beltrão de Andrade Lima, Emerson Luiz Lima Araújo, Lúcia Dias da Silva Guerra, Cláudia Polubriaginof, Ana Marcia de Sá Guimarães

**Affiliations:** 1 Universidade de São Paulo, Departamento de Enfermagem em Saúde Coletiva, São Paulo, SP, Brazil.; 2 Universidade Estadual de Campinas, Faculdade de Ciências Médicas, São Paulo, SP, Brazil.; 3 Universidade Federal do Ceará, Faculdade de Medicina, Altamira, CE, Brazil.; 4 Ministério da Saúde, Secretaria de Atenção Primária a Saúde, Brasília, DF, Brazil.; 5 Universidade de São Paulo, Faculdade de Saúde Pública, São Paulo, SP, Brazil.; 6 Faculdade de Ciências Médicas da Santa Casa de São Paulo, Programa de Pós-Graduação em Saúde Coletiva, São Paulo, SP, Brazil.; 7 Universidade de São Paulo, Instituto de Ciências Biomédicas, Departamento de Microbiologia, São Paulo, SP, Brazil.

**Keywords:** Tuberculosis, Ill-Housed Persons, Social Vulnerability, Socioeconomic Disparities in Health, South America, Tuberculose, Pessoas Mal Alojadas, Vulnerabilidade Social, Disparidades Socioeconômicas em Saúde, América do Sul

## Abstract

**Introduction:**

Tuberculosis (TB) remains a major public health problem in South America, disproportionately affecting vulnerable populations, such as the homeless population (HP).

**Objective:**

To synthesize, through a systematic review with meta-synthesis, the scientific evidence on the relationship between tuberculosis and its social, individual, and programmatic determinants in the HP in South America, highlighting factors associated with treatment abandonment and mortality.

**Methods:**

Scientific articles were searched in PubMed, EMBASE, LILACS, SciELO, Web of Science, and Cochrane databases, published in Portuguese, English, or Spanish between 2013 and 2023. Study selection was conducted independently by three reviewers, with consensus reached by a fourth when necessary. The process followed the protocols of the Cochrane Collaboration and the PRISMA flowchart, resulting in the inclusion of 27 studies.

**Results:**

The most frequent determinants were lack of social protection (85%) and extreme poverty (75%), both associated with high rates of treatment abandonment (average of 36%) and mortality (average of 14%). Reduced use of Directly Observed Therapy (DOT) and regional inequalities emerged as critical factors. The meta-synthesis indicated that alcohol and drug users had a 2.3 times higher risk of treatment abandonment, while TB-HIV coinfection increased mortality by up to threefold.

**Conclusion:**

The vulnerability of the HP to TB is strongly linked to social and programmatic factors. Intervention strategies should prioritize social protection, expansion of DOT, intersectoral policies, and approaches tailored to regional contexts in order to reduce inequalities and promote health equity.

## RESUMO

**Introdução:**

A tuberculose (TB) continua sendo um importante problema de saúde pública na América do Sul, afetando desproporcionalmente populações vulneráveis, como a população sem-teto (PST).

**Objetivo:**

Sintetizar, por meio de uma revisão sistemática com metassíntese, as evidências científicas sobre a relação entre tuberculose e seus determinantes sociais, individuais e programáticos no Sistema de Saúde da América do Sul, destacando os fatores associados ao abandono do tratamento e à mortalidade.

**Métodos:**

Foram pesquisados artigos científicos nas bases de dados PubMed, EMBASE, LILACS, SciELO, Web of Science e Cochrane, publicados em português, inglês ou espanhol entre 2013 e 2023. A seleção dos estudos foi realizada de forma independente por três revisores, sendo que, quando necessário, um quarto revisor chegou a um consenso. O processo seguiu os protocolos da Colaboração Cochrane e o fluxograma PRISMA, resultando na inclusão de 27 estudos.

**Resultados:**

Os determinantes mais frequentes foram a falta de proteção social (85%) e a pobreza extrema (75%), ambos associados a altas taxas de abandono do tratamento (média de 36%) e mortalidade (média de 14%). A redução no uso da Terapia Diretamente Observada (TDO) e as desigualdades regionais emergiram como fatores críticos. A metassíntese indicou que usuários de álcool e drogas apresentaram um risco 2,3 vezes maior de abandono do tratamento, enquanto a coinfecção por TB-HIV aumentou a mortalidade em até três vezes.

**Conclusão:**

A vulnerabilidade das PST à tuberculose está fortemente ligada a fatores sociais e programáticos. As estratégias de intervenção devem priorizar a proteção social, a expansão do TDO, políticas intersetoriais e abordagens adaptadas aos contextos regionais, a fim de reduzir as desigualdades e promover a equidade em saúde.

## INTRODUCTION

Tuberculosis (TB) is a respiratory infectious disease caused by *Mycobacterium tuberculosis*. It is a (disease) under viable diagnosis and cure within an appropriate care context, ranking among the ten leading causes of death worldwide and the primary cause of death from infectious respiratory diseases globally today^(1,2)^. In 2020, approximately six million people were affected by TB, with 90% being adults, 65% male, and 10% individuals living with the Human Immunodeficiency Virus (HIV)^(2)^. Over the past five years, the World Health Organization (WHO) has developed targets under the END TB Strategy, aiming to eliminate TB by reducing mortality by 95% and incidence by 90% between 2016 and 2035, compared to 2015 levels^(1,2)^.

In South America, responses to TB over the past two decades have shown moments of success, as evidenced by improved access to medications in some countries, such as Brazil, the incorporation of drug-resistant TB treatment into global public health policies, and innovations in diagnoses. Nevertheless, approximately 2 million people die globally from TB each year, with most fatalities occurring among young adults, and half of these cases involving TB/HIV coinfection^(3)^.

Although global incidence rates have decreased by approximately 2% annually in some regions^(1,2)^, this decline remains insufficient to meet the Sustainable Development Goals (SDGs) related to TB. Furthermore, 80% of confirmed cases are concentrated in 25 high-burden countries, with one-third occurring in India and China, where social determinants significantly contribute to disease dynamics^(2)^. Globally, about one billion people live in inadequate housing conditions, exacerbated by social instability, ethnic conflicts, and wars, factors likely to increase TB cases in the coming decades^(2,3)^. Among the key challenges in achieving the 17 SDG goals, addressing social inequalities stands out as a critical barrier to controlling TB^(4)^.

TB is a social determined disease, making it imperative to conduct this systematic review with a meta-synthesis approach, particularly for the homeless population. This group represents a heterogeneous demographic living in extreme poverty, with fragile or broken family ties, often using public spaces as temporary or permanent shelters^(5)^. They experience social vulnerabilities, including exposure to violence, substance abuse, unfavorable environmental conditions, pre-existing diseases like diabetes and hypertension, and sexually transmitted infections^(5)^. Based on current evidence, understanding the relationship between social inequality and TB can guide the development of more targeted and context-appropriate public health policies to address the disease in South America.

This study aims to analyze, through a systematic review with a meta-synthesis approach, the scientific evidence on the relationship between tuberculosis and its social, individual, and programmatic determinants among the homeless population in South America, focusing on factors associated with mortality and treatment abandonment, identifying gaps, and proposing strategies to address these vulnerabilities.

## METHODS

This is a systematic review with meta-synthesis^(6,7)^, aiming to describe the evidence on the relationship between tuberculosis (TB) and the determinants in the homeless population associated with TB-related mortality and treatment abandonment in South America through article analysis.

### Search Strategy, Information Sources, and Methodological Rigor

To address this objective, the following guiding question was proposed: *What is the scientific evidence on the relationship between tuberculosis, its determinants, and the outcomes of mortality and treatment abandonment in the homeless population in South America?*

A comprehensive bibliographic search was conducted in the PubMed, EMBASE, LILACS, SciELO, Web of Science, and Cochrane databases. Search strategies were adapted for each database, using controlled descriptors (MeSH/DeCS) and free-text keywords in English, Portuguese, and Spanish. The combined search strategy included terms related to “tuberculosis” and “homeless population”, along with country-specific filters for South America and epidemiological descriptors. An example of the search string was: *((“tuberculosis” OR “Mycobacterium tuberculosis”) AND (“homeless people*” OR “homeless population*” OR “street dwellers*” OR “homeless individuals*” OR “street population*”) AND (“Argentina” OR “Bolivia” OR “Brazil” OR “Chile” OR “Colombia” OR “Ecuador” OR “Guyana” OR “French Guiana” OR “Paraguay” OR “Peru” OR “Suriname” OR “Uruguay” OR “Venezuela”) AND (“epidemiology*” OR “prevalence*”))*.

### Eligibility Criteria

Studies were eligible if they were original scientific articles, available in full text, and published in Portuguese, English, or Spanish, between 2013 and 2023. This time frame was chosen because it encompasses the last decade, a period marked by significant advances in health policies targeting the homeless population and the prioritization of tuberculosis control in South America. Moreover, this interval includes publications aligned with WHO guidelines for tuberculosis control, ensuring updated and relevant evidence.

### Study Selection

Data collection and screening were conducted between April and June 2024. Three reviewers (EMR, CBS, and CP) independently evaluated the titles and abstracts of the retrieved studies. In cases of doubt or disagreement, a fourth reviewer (SBeL) was consulted to reach a final decision. All studies that met the eligibility criteria were read in full.

### Review Protocol

This systematic review was conducted in accordance with the protocols of the *Cochrane Collaboration*, which establish internationally recognized standards for high-quality reviews, ensuring methodological rigor, transparency in study selection, critical appraisal, and synthesis of evidence. In practice, we applied these recommendations as follows: (i) clear definition of the research question and eligibility criteria; (ii) comprehensive search across multiple international and regional databases; (iii) independent screening of titles and abstracts by three reviewers, with consensus reached through a fourth; (iv) full-text reading of eligible studies; and (v) documentation of the entire selection process through a PRISMA-adapted flowchart. This process ensured the traceability and reproducibility of the study.

### Quality Assessment

To minimize risk of bias, methodological quality of the included studies was assessed. Quantitative studies were evaluated using the Strengthening the Reporting of Observational Studies in Epidemiology (STROBE) checklist and were classified as: A (≥80% of criteria met), B (50–79%), or C (<50%, excluded from synthesis). Qualitative studies were assessed with the Critical Appraisal Skills Programme (CASP) tool, with scoring based on fulfillment of each criterion: 1 point (fully met), 0.5 points (partially met), and 0 points (not met). Scores ranged from 0–10 and were categorized as high rigor (≥9 points), moderate rigor (5–8.5), and low rigor (<5, excluded from meta-synthesis).

### Registration

This review was prospectively registered in the PROSPERO database on May 13, 2024, under registration number CRD42024541613.

### Eligibility Criteria, Analysis of Reviewed Articles, and Triangulation Methodology

#### Study Selection and Analysis Process

**Figure 1:**
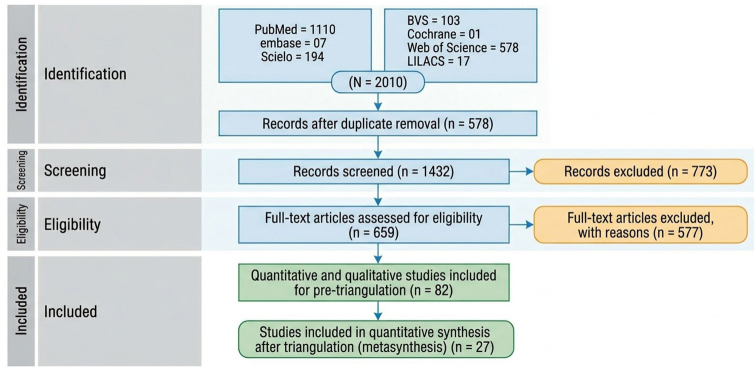
Search strategy for the studies included in the research.

The searches identified a total of 2.010 records, which were imported into the Rayyan software for initial screening. In the first stage, 578 duplicates were removed. Subsequently, three reviewers independently screened the titles and abstracts, and 773 studies were excluded for being out of scope. After this step, 659 articles were assessed in full text. Of these, 577 were excluded for not meeting the eligibility criteria, resulting in 82 potentially relevant articles. Following detailed analysis, 27 studies were included in the final meta-synthesis (Figure 1).

Based on: Moher et al.^(8)^. For more information, visit the PRISMA website^(9)^

#### Eligibility Criteria

Original studies were included if they: (i) addressed tuberculosis in the homeless population in South America; (ii) analyzed social, individual, or programmatic determinants; and (iii) reported outcomes related to mortality, adherence, or treatment abandonment. Articles were excluded if they: (i) did not focus on the homeless population; (ii) were limited to descriptive prevalence or risk estimates without determinant analysis; (iii) assessed only drug effectiveness without considering broader social determinants; or (iv) were editorials, narrative reviews, or case reports.

#### Meta-Synthesis and Triangulation

The analysis of the 27 studies followed the meta-synthesis method, structured around three pillars:

Meta-theory: assessment of the conceptual framework underlying the studies, addressing themes such as access to health services, street living conditions, food insecurity, social stigma, drug resistance, treatment abandonment, and mortality^(10,11)^.

Meta-method: examination of the coherence of methodological designs, highlighting strengths and limitations of the strategies used and their ability to generate consistent and relevant evidence^(11,12)^.

Qualitative meta-analysis: thematic integration of results, identifying determinants, vulnerabilities, and clinical and epidemiological outcomes^(11,13)^.

Methodological triangulation was applied to integrate quantitative and qualitative findings. The following aspects were reviewed for data extraction: study title, study type, vulnerability factors, and main outcomes^(14,15)^.

Convergence was considered when two or more studies presented similar patterns, such as the influence of extreme poverty, substance use, and social stigma on treatment abandonment^(14)^. Divergence reflected regional, contextual, and methodological variations, such as the differing prevalence of TB-HIV coinfection across countries, highlighting the need for tailored interventions adapted to specific local contexts^(15)^.

## RESULTS

Chart 1 highlights the vulnerability of the homeless population to tuberculosis (TB) in South America, reporting therapeutic failure rates of 38.63%. Silva et al.^(16)^ reported that in Brazil, 14.059 individuals registered in the Health Information Systems (*SINAN*) exhibited a treatment abandonment rate of 35.2% (p < 0.05). In Roraima, Soares et al.^(17)^ identified abandonment rates exceeding 40% (standard deviation ±5.1), with the highest impact on the immigrant population. Cross-sectional studies, such as that by Gioseffi et al.^(18)^, emphasized a greater likelihood of abandonment among alcohol and drug users (odds ratio 2.3; 95% CI, 1.8–2.9). Additionally, Ranzani et al.^(19)^ described treatment failure in 57.3% of cases in the homeless population, with high mortality associated with TB-HIV coinfection.

**Chart 1:** Overview of included studies.

Authors	Study Type	Location in South America	Population	Analyzed Variable	Programmatic Vulnerability	Outcomes
Gioseffi et al.^(18)^	Cross-sectional.	Brazil.	Homeless Population in the Municipality of Rio de Janeiro.	Gender, race/color, HIV, type of TB, alcohol consumption, tobacco, illicit drugs, DOT, notification district.	High population density, socioeconomic barriers, stigma, and lack of social protection.	High treatment dropout rate and greater likelihood of abandonment among alcohol and drug users.
Macedo et al.^(34)^	Descriptive logistic regression analysis.	Brazil.	Incarcerated individuals (53.6%) (PPL) and the homeless population (54.5%) (PSR).	Cure, treatment abandonment, TB-related death, other causes, MDR-TB, treatment regimen changes, education level, comorbidities (AIDS, diabetes, and mental illness), alcohol, tobacco, and illicit drugs.	Poverty, lack of social protection, economic and social barriers.	The homeless population (HP) shows higher rates of treatment failure and TB-related mortality, while incarceration (PPL) acts as a protective factor against failure.
Cadorin and Maggi^(35)^	Cross-sectional, quantitative approach.	Brazil.	18 reported cases in the homeless population (HP) in Acre.	Incidence, prevalence, contacts examined, type of entry, sputum culture, HIV, and treatment outcomes.	Poverty, lack of social programs, and lack of social protection.	There is a high cure rate (50%), abandonment rate (16.7%), and mortality rate (11.1%).
Soares et al.^(17)^	Quantitative, cross-sectional, and descriptive.	Brazil.	Confirmed TB cases in the state of Roraima from 2009 to 2019.	Socio-demographic profile, including clinical characteristics, diagnosis, and case follow-up.	Poverty, lack of social protection, and greater exposure to adverse conditions for migrants.	Migrants in the homeless population (HP) have higher dropout rates and lower treatment success, highlighting significant inequalities.
Rodrigues^(36)^	Descriptive, quantitative.	Brazil.	Homeless population in Brazil in the year 2022.	Gender, age, HIV infections, smoking, alcoholism, comorbidity, and treatment outcomes.	Stigma, poverty, lack of social protection.	High treatment dropout rate (181 cases) and the presence of DOT in confirmed AIDS cases.
Carneiro et al.^(37)^	Cross-sectional, observational with a quantitative approach.	Brazil.	The homeless population in the municipality of Passos (MG), with 78 interviews.	Gender, age, education level, place of sleep, duration of homelessness source of income, contacts with TB, use of alcohol, tobacco, illicit drugs, comorbidities, and access to hygiene.	Poverty, lack of social protection, nomadism, stigma, and breakdown of family ties.	Not specified. It reports that the high prevalence of risk factors increases vulnerability to TB.
Santos et al.^(14)^	Cross-sectional with a quantitative approach.	Brazil.	1530 TB patients experiencing homelessness registered in CadÚnico.	Gender and race/color.	Experiencing homelessness and extreme poverty.	High treatment failure rate, evidenced by a high percentage of treatment abandonment and a significant mortality rate, along with a relatively low probability of cure compared to the general population.
Ranzani et al.^(38)^	Longitudinal with a quantitative approach.	Brazil.	Population with tuberculosis in the state of São Paulo (2010 to 2015).	Gender, age, and race/color.	Social factors, health habits, and comorbidities.	A high cure rate, but still with significant proportions of interventions needed to improve follow-up and reduce social and health vulnerabilities affecting these patients.
Ranzani et al.^(19)^	Quantitative, retrospective.	Brazil	Homeless population with pulmonary TB in the state of São Paulo (2009–2013).	Gender, skin color, alcohol and drug use, type of tuberculosis, HIV, diabetes, and treatment outcomes.	Not mentioned.	Treatment failure rate of 57.3%, characterized by high rates of abandonment and mortality.
Snyder et al.^(20)^	Quantitative, retrospective.	Brazil.	People living in a family or non-family groups, experiencing homelessness, reported TB in 2010.	Gender, type of tuberculosis, HIV, treatment outcomes (Cure and mortality).	Not mentioned.	The use of DOT (Directly Observed Therapy) led to an increase in cure rates to 54% and a reduction in treatment abandonment rates to 16% and mortality rates to 6% among the population living in informal settlements and the homeless population.
Rodrigues et al.^(21)^	Quantitative, retrospective	Brazil.	Homeless population with tuberculosis (2015–2020).	Gender, age, race/color, smoking, alcoholism, drug abuse, HIV, diabetes, type of tuberculosis, outcomes, and treatment (cure, mortality, and abandonment)	Not mentioned.	Treatment failure in 57.3% of cases of abandonment and mortality. The main predictors were drug use and the absence of DOT.
Aguiar et al.^(40)^	Quantitative, descriptive, and cross-sectional.	Brazil.	Incarcerated individuals and homeless people with TB (2015–2017).	Gender, age, race/color, education level, HIV, alcoholism, smoking, types of tuberculosis, treatment outcomes.	Deficient healthcare system, and lack of social protection.	Treatment abandonment is the main outcome in the homeless population (HP), with low use of molecular testing and DOT.
Sousa et al.^(45)^	Qualitative, descriptive and exploratory.	Brazil.	People above 18 years living with HIV/AIDS coinfected with tuberculosis in 2011.	Use of alcohol, illicit drugs, adherence to treatment, social and economic difficulties related to the life styleof the homeless.	Not mentioned.	Social and economic barriers and life style that hinder treatment adherence, abandonment rate of 7.6%.
Ranzani et al.^(43)^	Epidemiological and descriptive with trend analysis and modeling.	Brazil, Argentina, Chile, Colombia, Ecuador, El Salvador, Peru, Uruguay, and Venezuela.	Age, gender, and people with HIV stratify the general population.	Age, gender, increase in TB incidence, economic and social factors.	Deficient healthcare system, and lack of social protection.	Increasing rates of incidence, mortality, abandonment, and challenges in regional control of tuberculosis in each South American country.
Maffacciolli et al.^(25)^	Qualitative, descriptive, and exploratory.	Brazil.	12 people in the homeless population (6 men and 6 women) with TB.	Hospitalization trajectories, life history, drug use, and dynamics of affective-sexual relationships.	Deficient healthcare system in providing care to the homeless population (HP).	Identification of associated social, economic, and health vulnerabilities
Pavinati et al.^(26)^	Descriptive, ecological, geospatial.	Brazil.	Adults in the homeless population (HP) with tuberculosis.	Epidemiological indicators, treatment outcomes, regional and temporal disparities.	Deficient healthcare system.	High abandonment rates and mortality, regional inequalities in the performance of epidemiological indicators.
Freitas et al.^(27)^	Quantitative and descriptive.	Brazil	21.841 TB cases (21.305 in the general population, 349 in the incarcerated population, and 187 in the homeless population).	Rapid molecular test (RMT), time between diagnosis and treatment initiation, HIV testing rate, DOT, cure, and treatment abandonment.	Lack of social protection, missing or incorrect data, low utilization of the RMT.	DOT performed in only 22% of the homeless population (HP), with high rates of abandonment and mortality.
Zuim and Trajman^(28)^	Qualitative and interpretive.	Brazil.	19 adult patients in the homeless population (HP) with TB.	Gender, age, race/color, nutrition, alcohol use, drug abuse, HIV, fragmentation of care, and stigma.	Deficient healthcare system and lack of social protection.	Stigma, treatment abandonment, TB-related mortality, and deficiencies in continuous care.
Lima et al.^(29)^	Descriptive, exploratory, and Quantitative.	Brazil.	70 individuals are experiencing homelessness.	Gender, age group, race/color, education level, smoking, alcoholism, use of public health services, poverty, sputum smear results.	Deficient healthcare system and lack of social protection.	High abandonment rate in the prevalence of abandonment and social vulnerability to TB.
Fica et al.^(30)^	Descriptive, cross-sectional.	Chile.	General population and special groups (homeless population).	Gender, age, HIV/AIDS, alcohol and drug use, poverty, indicators of the TB control program (PROCET).	Deficient healthcare system and lack of modernization in diagnosis.	Increase in TB incidence, high lethality (>3%), loss to follow-up (>5%), low cure rate (<90%), and increase in TB among the homeless population.
Sales et al.^(42)^	Ecological and descriptive.	Brazil.	Analyzed 577 census sectors (588 TB cases between 2009 and 2011).	Gender, age, education, income, access to basic sanitation, underreporting of cases, and spatial distribution.	Deficient healthcare system, low epidemiological surveillance, and high underreporting.	Identification of underreporting in 93% of sectors with no reported cases and higher TB prevalence in low-income and low-education areas.
Pinheiro et al.^(31)^	Correlational analysis.	Brazil.	144,953 individuals from nine metropolitan regions of Brazil, PNAD (National Household Sample Survey) 2008.	Demographic (gender, age, socioeconomic status), health conditions, access to healthcare services.	Limited availability of healthcare services in some metropolitan regions of Brazil.	A high correlation between self-reported tuberculosis and official notification data.
Pinheiro et al ^(22)^	Integrative literature review	Brazil	Homeless population (people experiencing homelessness) with tuberculosis.	Care trajectories; care practices; treatment adherence; role of Street Outreach Clinics (Consultório na Rua – CnaR); social determinants (substance use, housing instability, food insecurity).	High programmatic and social vulnerability: barriers to healthcare access, discontinuity of care, fragmentation of health services, institutional stigma, social invisibility, weak intersectoral coordination.	Low adherence associated with adverse social conditions; treatment abandonment influenced by substance use and unstable living conditions; improved adherence with integrated and tailored care approaches; CnaR as a key device for building trust and ensuring comprehensive care.
Hino et al.^(32)^	Qualitative, thematic oral history.	Brazil.	24 homeless individuals (HP) undergoing TB treatment.	Life experience, treatment adherence, perception of healthcare services.	Deficient healthcare system, and lack of social protection.	Treatment obstacles associated with street life, perspectives of cure as a motivator for treatment adherence.
Silva et al.^(33)^	Ecological.	Brazil.	2966 TB cases (544 cases of abandonment).	Age group, gender, race/color, education, HIV, type of TB, and associated health conditions (alcohol, drugs).	Deficient healthcare system, lack of social protection, barriers to access to treatment.	18.7% treatment abandonment, with a progressive increase in specific years.
Rodrigues and Tauil^(41)^	Epidemiological and ecological.	Brazil.	New TB cases from 2006 to 2015.	Gender, age, HIV, smoking, alcoholism, diabetes, illicit drugs, clinical form.	Deficient healthcare system.	Reduction in TB incidence, declining cure rate, increase in abandonment over time.
Silva et al.^(16)^	Descriptive and cross-sectional study.	Brazil.	Homeless population (HP) in CadÚnico (2014 to 2019).	Gender, age group, race/color, education level, income, lack of social protection, and inequality in access.	Deficits in healthcare assistance and lack of social protection.	High abandonment rate and low treatment coverage.

Source: Authors, 2024.

Directly Observed Therapy increases cure rates from 54% to 67% (p < 0.05) in the homeless population, according to Snyder et al.^(20)^ (Table 1). Data Triangulation of the Analyzed Studies in summary materials). The analysis of Table 1 highlights the convergent and divergent aspects related to the socioeconomic profile, treatment outcomes, programmatic vulnerabilities, geographical distribution, and social income/benefits in South America’s homeless population with TB.

In the socioeconomic profile, all studies converged on the predominance of men aged 20 to 49, with low educational levels and a high proportion of Black and mixed-race individuals, considering racial and educational vulnerabilities as risk factors. The average convergence in this aspect was 74%. Regarding treatment outcomes, high dropout rates were described in studies such as Rodrigues et al.^(21)^, with 36.02% dropout and 14.37% mortality, while Pinheiro et al.^(22, 31)^ reported that the cure rate decreased from 39.6% to 34.5% between 2014 and 2018 in the region. The average convergence was 68%. However, few studies (n = 09) explored strategies to reverse the increase in dropouts, such as interventions based on DOT. In programmatic vulnerability, 65% of the studies reported insufficient social protection as a determining factor, with low coverage of social programs such as *Bolsa Família* (4% in Silva et al.^(16)^).

However, no consensus was described regarding the implementation of intersectoral policies to mitigate the impacts of extreme poverty on treatment adherence. The average convergence was 67%. Geographical distribution highlighted a concentration in urban areas of South America, with some regions such as the North and Northeast remaining underrepresented. Only 12% of the studies described the geographic mobility of the homeless population as a barrier to care, emphasizing the need for health policies tailored to remote and border areas.

Finally, concerning income and social benefits, extreme poverty predominated in 90% of the homeless populations analyzed in the studies, but the direct impacts of social programs on improving outcomes remain poorly evaluated. The average convergence was 72%.

The analysis of the studies reveals that 12% addressed the role of temporary housing instability, such as the use of public shelters, as an intermediate factor in treatment adherence and clinical follow-up. Furthermore, less than 10% discussed the influence of cultural factors on the acceptance of therapeutic interventions among subgroups of the homeless population, raising the need for approaches that address the heterogeneity of this population. This analysis underscores the importance of integrating longitudinal and contextual data to better understand the social dynamics affecting health outcomes in the homeless population in South America.

**Figure 2:**
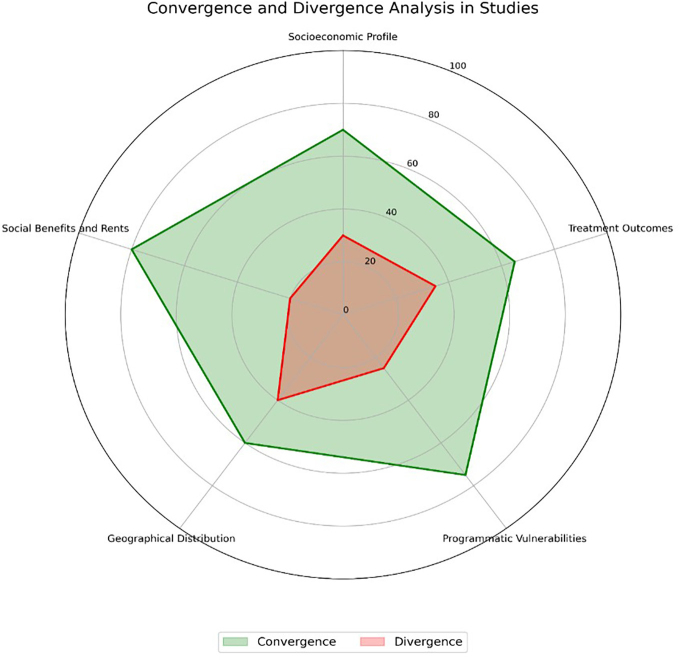
Convergence and divergence in tuberculosis among the homeless population in South America, 2024.

**Source:** Authors, 2024.

In Figure 2, the relationship between the geographical distribution of homeless populations and the divergences in programmatic Vulnerabilities for TB treatment, particularly in the context of DOT, is highlighted. Although DOT is used as an effective strategy in the analyzed studies, incomplete convergence (75%) describes specific barriers to its implementation, such as the difficulty in maintaining continuity of care among frequently mobile populations like the homeless.

Another relevant aspect is the impact of transitional social programs, such as emergency aid, on treatment adherence in the homeless population. The high convergence on social benefits (80%) contrasts with the absence of studies evaluating how these temporary policies affect long-term treatment continuity, particularly in densely populated urban areas. Additionally, the relationship between climatic factors and adherence to DOT remains a neglected gap in the literature. Studies such as those by Soares et al.^(17)^ suggest regional challenges but do not directly connect seasonality to therapeutic outcomes. In the analysis of border regions, such as Roraima, the challenges posed by intense rainfall, which hinder access to health services for the homeless population, are emphasized, as reported by Soares et al.^(17)^

**Figure 3:**
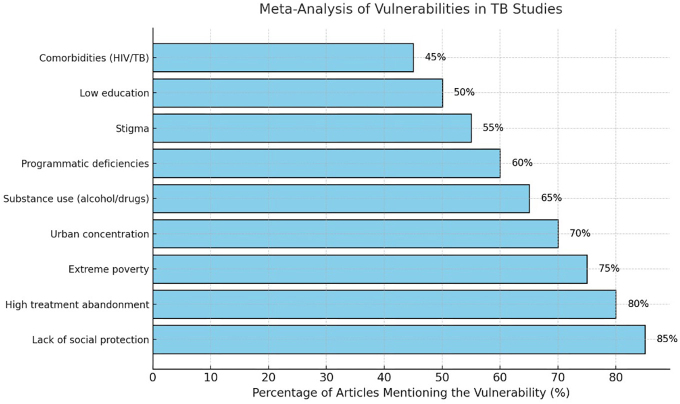
Meta-analysis of vulnerabilities in studies on TB in the homeless population.

**Source:** Authors, 2024.

In Figure 3, the lack of social protection (85%) stands out as the primary determinant identified in the analyzed studies, followed by treatment abandonment (80%) and extreme poverty (75%). Urban concentration (70%) and illicit drug use (65%) are also highlighted. Other vulnerabilities, such as programmatic deficiencies (60%), stigma (55%), low education levels (50%), and TB-HIV comorbidities (45%), reflect persistent social and structural barriers. In Figure 2, social and programmatic vulnerabilities show an average of 1.75 (95% CI: 1.50–2.00), indicating the strong impact of this determinant on tuberculosis outcomes.

In Figure 2, Geographical vulnerabilities exhibit a moderate effect, with an average of 1.10 (95% CI: 1.05–1.20), characterizing regional inequalities as influential factors in TB treatment in the region. Social benefits, on the other hand, have a smaller impact, with an average of 1.05 (95% CI: 1.00–1.15), suggesting insufficient coverage and implementation. Treatment outcomes, such as abandonment and mortality, showed an effect size of 1.25 (95% CI: 1.15–1.35), reinforcing the influence of social and structural determinants on TB treatment responses.

In Figure 2 and 3, there is a description of the relative risk (RR) associated with social vulnerabilities in the homeless population with tuberculosis. HIV coinfection presents the highest relative risk (RR = 3.0, 95% CI: 2.5–3.5), followed by illicit drug use (RR = 2.5, 95% CI: 2.0–3.0) and alcoholism (RR = 2.0, 95% CI: 1.7–2.5). In Figure 3, housing instability and extreme poverty also increase risk, with RRs of 1.8 (95% CI: 1.4–2.2) and 1.7 (95% CI: 1.5–2.0), respectively. Limited access to DOT and insufficient coverage of social benefits show moderate RRs, around 1.5 (95% CI: 1.2–1.8), highlighting priority areas for interventions.

## DISCUSSION

The results obtained from the analysis of the 27 included studies highlight different determinants of TB-associated vulnerability in the HP in South America. These findings reveal the intersection of individual, social, and programmatic vulnerabilities, emphasizing factors that perpetuate inequalities in access to diagnosis, treatment, and favorable outcomes^(15,23,24,25,26,27)^. In the programmatic context, the limited implementation of policies aimed at the HP, such as the *Consultórios na Rua* (Street Clinics), with only 60% of the projected teams active by 2019, indicates the need for greater governmental prioritization in Brazil. Reforms in information systems and the strengthening of support networks emerge as essential measures to improve care, particularly in regions with a higher concentration of Vulnerabilities^(15,23,24,25,26,27)^.

Regarding individual vulnerabilities, the analyzed studies highlight the influence of the use of illicit drugs, alcohol, and other substances on adherence to TB treatment. This association was linked to treatment dropout rates of up to 33% in some regions of Brazil and other South American countries, with a relative risk 2.5 times higher among substance users, a value seven times above the acceptable threshold defined by WHO^(16,18,28,29,30,31,32,33)^.

Constant mobility, nomadism, and the breakdown of family ties increase barriers to accessing healthcare services and compromise continuity of care, while comorbidities such a mental disorders and TB-HIV coinfection exacerbate the challenges faced by the HP^(16,18,34,35)^. TB-HIV coinfection, reported in 45% of the studies, reflects the complexity of these determinants, being associated with increased mortality and the intensification of social stigma^(16,18,34,35)^.

The predominant profile identified, with a mean age of 49.8 years and higher prevalence among young adult men, also suggests greater vulnerability associated with the mobility of this population^(16,18,34,35)^. In terms of social vulnerabilities, precarious living conditions and systemic exclusion reinforce structural inequalities. The studies showed that 63.6% of individuals belonged to Black or mixed-race groups, highlighting how racial and economic disparities reinforce marginalization, particularly in Brazil^(16,18,32,33)^.

A history of incarceration, recurrently reported across publications, indicates prolonged cycles of social exclusion, in which recently released individuals face stigma, lack of economic opportunities, and insufficient institutional support, often culminating in homelessness^(16,18,34,35)^. Extreme poverty, identified in 75% of the publications, further hinders access to basic health services and social assistance programs, while the low coverage of social benefits such as *Bolsa Família*, especially in the North and Northeast regions of Brazil, constitutes a critical barrier to TB control among the HP^(34,35)^.

Programmatic vulnerabilities were also widely evidenced. Studies indicated that 57% of TB cases in the HP resulted in unfavorable outcomes, with drug use (OR = 1.54; 95% CI: 1.31–1.80) and the absence of supervised treatment (OR = 0.52; 95% CI: 0.45–0.60) significantly increasing the risk of treatment dropout and mortality^(16,18,34,35)^. In border regions, such as Roraima, limited healthcare infrastructure combined with climatic and logistical barriers compromised timely diagnosis and treatment^(16,18,34,35)^. The limited coverage of the Rapid Molecular Test (RMT-TB), especially in large urban centers such as São Paulo, Belo Horizonte, and Rio de Janeiro, illustrates critical failures in case management^(17,36,37)^.

Analyses also indicated that health policies, often disconnected from local realities, do not fully incorporate social, economic, and environmental determinants that influence outcomes for the HP^(17,36,37)^. This limitation is aggravated by the low coverage of DOT, which is directly associated with increased dropout and mortality^(17,20,36,37,38)^.

Despite advances, significant gaps persist. Temporary housing instability, such as shelter stays, was poorly explored as an intermediate factor for treatment adherence. Moreover, climatic and environmental factors remain largely neglected, despite their potential influence on TB incidence and management, as observed in border regions affected by heavy rainfall^(19,21,39,40,41)^.

The cultural and regional diversity of South America also demands context-specific analyses that recognize local particularities. In this scenario, intersectoral actions integrating health policies with social programs, such as the expansion of social protection benefits, are essential to mitigating extreme poverty and reducing inequalities^(19,21,39,40,41)^. Such initiatives must align with the 2030 Agenda and the SDGs, ensuring health as a basic human right and promoting equity through the integration of social protection, health technologies, and interventions adapted to territorial and climatic contexts^(22,42,43,44,45,46,47)^.

## CONCLUSION

The analysis of the 27 included studies highlights the multiple vulnerabilities faced by the HP in South America in the context of tuberculosis. The predominant profile was characterized by young men, with a higher proportion of Black and mixed-race individuals, low educational levels, and living in extreme poverty, reflecting persistent structural inequalities that directly impact clinical and epidemiological outcomes.

The high average rates of treatment abandonment (36%) and mortality (14%) are strongly associated with social and programmatic barriers, such as insufficient coverage of social benefits (85%) and the limited implementation of protection programs, such as *Bolsa Família*. Geographic analysis also revealed the underrepresentation of remote and border regions, where population mobility and adverse climatic conditions increase difficulties in timely access to healthcare services.

Although DOT is recognized as an effective strategy in Brazil, its coverage remains marked by regional inequalities and structural limitations, compromising adherence and treatment success. Aspects such as temporary housing instability and the influence of climatic factors on treatment outcomes were scarcely explored, indicating the need for longitudinal analyses that integrate social, environmental, and programmatic determinants. In light of these findings, the formulation and expansion of intersectoral public policies, built on the principle of equity and adapted to the specific needs of the HP, are urgently required.

The incorporation of measures to strengthen social protection, expand DOT coverage, integrate diagnostic technologies, and consider territorial and climatic factors is essential to address regional inequalities and promote sustainable improvements in the health indicators of the homeless population in South America.

## Data Availability

The entire dataset supporting the results of this study was published in the article itself.
