# Supplementary material for: Tuberculosis in the South America’s homeless: a systematic review of social determinants, control strategies and health equity
Source: Rev Esc Enferm USP. 2026 May 1;60:e20250222. doi: 10.1590/1980-220X-REEUSP-2025-0222en (PMC13159549; doi:10.1590/1980-220X-REEUSP-2025-0222en)
Supplement: Translation

# Translation: Tuberculosis en personas sin hogar de América del Sur: una revisión sistemática de determinantes sociales, estrategias de control y equidad en salud

**DOI:** 10.1590/1980-220X-REEUSP-2025-0222es

**Authors:** Eliza Miranda Ramos, Carolina Braga Sisti, Sérgio Beltrão de Andrade Lima, Emerson Luiz Lima Araújo, Lúcia Dias da Silva Guerra, Cláudia Polubriaginof, Ana Marcia de Sá Guimarães

**Affiliations:** 1 Universidade de São Paulo, Departamento de Enfermagem em Saúde Coletiva, São Paulo, SP, Brazil.; 2 Universidade Estadual de Campinas, Faculdade de Ciências Médicas, São Paulo, SP, Brasil.; 3 Universidade Federal do Ceará, Faculdade de Medicina, Altamira, CE, Brasil.; 4 Ministério da Saúde, Secretaria de Atenção Primária a Saúde, Brasília, DF, Brasil.; 5 Universidade de São Paulo, Faculdade de Saúde Pública, São Paulo, SP, Brasil.; 6 Faculdade de Ciências Médicas da Santa Casa de São Paulo, Programa de Pós-Graduação em Saúde Coletiva, São Paulo, SP, Brasil.; 7 Universidade de São Paulo, Instituto de Ciências Biomédicas, Departamento de Microbiologia, São Paulo, SP, Brasil.

**Keywords:** Tuberculosis, Personas con Mala Vivienda, Vulnerabilidad Social, Disparidades Socioeconómicas en Salud, América del Sur

## Abstract

**Introducción:**

La tuberculosis (TB) sigue siendo un importante problema de salud pública en América del Sur, que afecta desproporcionadamente a las poblaciones vulnerables, como la población sin hogar (PS).

**Objetivo:**

Sintetizar, mediante una revisión sistemática con metasíntesis, la evidencia científica sobre la relación entre la tuberculosis y sus determinantes sociales, individuales y programáticos en la PS de Sudamérica, destacando los factores asociados al abandono del tratamiento y la mortalidad.

**Métodos:**

Se buscaron artículos científicos en las bases de datos PubMed, EMBASE, LILACS, SciELO, Web of Science y Cochrane, publicados en portugués, inglés o español entre 2013 y 2023. La selección de los estudios fue realizada de forma independiente por tres revisores y, cuando fue necesario, un cuarto revisor llegó a un consenso. El proceso siguió los protocolos de la Colaboración Cochrane y el diagrama de flujo PRISMA, dando como resultado la inclusión de 27 estudios.

**Resultados:**

Los determinantes más frecuentes fueron la falta de protección social (85%) y la pobreza extrema (75%), ambos asociados con altas tasas de abandono del tratamiento (promedio de 36%) y mortalidad (promedio de 14%). El uso reducido de la terapia de observación directa (TOD) y las desigualdades regionales surgieron como factores críticos. La metasíntesis indicó que los usuarios de alcohol y drogas tenían un riesgo 2,3 veces mayor de abandonar el tratamiento, mientras que la coinfección TB-VIH aumentaba la mortalidad hasta tres veces.

**Conclusión:**

La vulnerabilidad de los PS a la tuberculosis está fuertemente vinculada a factores sociales y programáticos. Las estrategias de intervención deben priorizar la protección social, la expansión de la TOD, las políticas intersectoriales y los enfoques adaptados a los contextos regionales para reducir las desigualdades y promover la equidad en salud.

## INTRODUCCIÓN

La tuberculosis (TB) es una enfermedad infecciosa respiratoria causada por *Mycobacterium tuberculosis*. Se trata de una enfermedad diagnosticable y curable en un contexto de atención adecuada, que se encuentra entre las diez principales causas de muerte en todo el mundo y es la principal causa de muerte por enfermedades respiratorias infecciosas a nivel mundial en la actualidad^(1,2)^. En 2020, aproximadamente seis millones de personas se vieron afectadas por la TB, de las cuales el 90% eran adultos, el 65% hombres y el 10% personas que vivían con el virus de la inmunodeficiencia humana (VIH)^(2)^. En los últimos cinco años, la Organización Mundial de la Salud (OMS) ha desarrollado objetivos en el marco de la Estrategia END TB, con el fin de eliminar la tuberculosis reduciendo la mortalidad en un 95% y la incidencia en un 90% entre 2016 y 2035, en comparación con los niveles de 2015^(1,2)^.

En América del Sur, las respuestas a la tuberculosis durante las últimas dos décadas han tenido momentos de éxito, como lo demuestra la mejora del acceso a los medicamentos en algunos países, como Brasil, la incorporación del tratamiento de la tuberculosis resistente a los medicamentos en las políticas de salud pública mundiales y las innovaciones en el diagnóstico. No obstante, aproximadamente 2 millones de personas mueren cada año en todo el mundo a causa de la tuberculosis, la mayoría de ellas jóvenes adultos, y la mitad de estos casos son de coinfección por tuberculosis y VIH^(3)^.

Aunque las tasas de incidencia mundiales han disminuido aproximadamente un 2% anual en algunas regiones^(1,2)^, este descenso sigue siendo insuficiente para cumplir los Objetivos de Desarrollo Sostenible (ODS) relacionados con la tuberculosis. Además, el 80% de los casos confirmados se concentran en 25 países con alta carga de morbilidad, y un tercio de ellos se producen en la India y China, donde los determinantes sociales contribuyen de manera significativa a la dinámica de la enfermedad^(2)^. A nivel mundial, alrededor de mil millones de personas viven en condiciones de vivienda inadecuadas, agravadas por la inestabilidad social, los conflictos étnicos y las guerras, factores que probablemente aumentarán los casos de tuberculosis en las próximas décadas^(2,3)^. Entre los principales retos para alcanzar los 17 objetivos de los ODS, la lucha contra las desigualdades sociales destaca como una barrera fundamental para el control de la tuberculosis^(4)^.

La tuberculosis es una enfermedad determinada por factores sociales, por lo que es imperativo realizar esta revisión sistemática con un enfoque de metásíntesis, en particular para la población en situación de calle. Este grupo representa una población heterogénea que vive en condiciones de extrema pobreza, con lazos familiares frágiles o rotos, y que a menudo utiliza los espacios públicos como refugios temporales o permanentes^(5)^. Sufren vulnerabilidades sociales, como la exposición a la violencia, el abuso de sustancias, condiciones ambientales desfavorables, enfermedades preexistentes como la diabetes y la hipertensión, e infecciones de transmisión sexual^(5)^. Según las pruebas actuales, comprender la relación entre la desigualdad social y la tuberculosis puede orientar el desarrollo de políticas de salud pública más específicas y adecuadas al contexto para abordar la enfermedad en América del Sur.

El objetivo de este estudio es analizar, mediante una revisión sistemática con un enfoque de metásíntesis, la evidencia científica sobre la relación entre la tuberculosis y sus determinantes sociales, individuales y programáticos entre la población en situación de calle en América del Sur, centrándose en los factores asociados con la mortalidad y el abandono del tratamiento, identificando las lagunas y proponiendo estrategias para abordar estas vulnerabilidades.

## MÉTODOS

Se trata de una revisión sistemática con metásíntesis^(6,7)^, cuyo objetivo es describir la evidencia sobre la relación entre la tuberculosis (TB) y los determinantes en la población en situación de calle asociados con la mortalidad relacionada con la TB y el abandono del tratamiento en América del Sur a través del análisis de artículos.

### Estrategia de Búsqueda, Fuentes de Información y Rigor Metodológico

Para abordar este objetivo, se propuso la siguiente pregunta orientativa: *¿Cuál es la evidencia científica sobre la relación entre la tuberculosis, sus determinantes y los resultados de mortalidad y abandono del tratamiento en la población en situación de calle de América del Sur?*

Se realizó una búsqueda bibliográfica exhaustiva en las bases de datos PubMed, EMBASE, LILACS, SciELO, Web of Science y Cochrane. Las estrategias de búsqueda se adaptaron a cada base de datos, utilizando descriptores controlados (MeSH/DeCS) y palabras clave de texto libre en inglés, portugués y español. La estrategia de búsqueda combinada incluyó términos relacionados con «tuberculosis» y «población en situación de calle», junto con filtros específicos por país para América del Sur y descriptores epidemiológicos. Un ejemplo de la cadena de búsqueda fue: *((“tuberculosis” OR “Mycobacterium tuberculosis”) AND (“homeless people*” OR “homeless population*” OR “street dwellers*” OR “homeless individuals*” OR “street population*”) AND (“Argentina” OR “Bolivia” OR “Brasil” OR “Chile” OR “Colombia” OR “Ecuador” OR “Guyana” OR “French Guiana” OR “Paraguay” OR “Peru” OR “Suriname” OR “Uruguay” OR “Venezuela”) AND (“epidemiology*” OR “prevalence*”))*

### Criterios de Elegibilidad

Los estudios eran elegibles si eran artículos científicos originales, disponibles en texto completo y publicados en portugués, inglés o español entre 2013 y 2023. Se eligió este periodo porque abarca la última década, un periodo marcado por avances significativos en las políticas de salud dirigidas a la población en situación de calle y la priorización del control de la tuberculosis en América del Sur. Además, este intervalo incluye publicaciones alineadas con las directrices de la OMS para el control de la tuberculosis, lo que garantiza una evidencia actualizada y relevante.

### Selección de Estudios

La recolección y selección de datos se llevó a cabo entre abril y junio de 2024. Tres revisores (EMR, CBS y CP) evaluaron de forma independiente los títulos y resúmenes de los estudios recuperados. En caso de duda o desacuerdo, se consultó a un cuarto revisor (SBeL) para llegar a una decisión final. Se leyeron íntegramente todos los estudios que cumplían los criterios de elegibilidad.

### Protocolo de Revisión

Esta revisión sistemática se llevó a cabo de acuerdo con los protocolos de la *Cochrane Collaboration*, que establecen normas reconocidas internacionalmente para revisiones de alta calidad, lo que garantiza el rigor metodológico, la transparencia en la selección de estudios, la evaluación crítica y la síntesis de la evidencia. En la práctica, aplicamos estas recomendaciones de la siguiente manera: (i) definición clara de la pregunta de investigación y los criterios de elegibilidad; (ii) búsqueda exhaustiva en múltiples bases de datos internacionales y regionales; (iii) selección independiente de títulos y resúmenes por parte de tres revisores, con consenso alcanzado a través de un cuarto; (iv) lectura del texto completo de los estudios elegibles; y (v) documentación de todo el proceso de selección mediante un diagrama de flujo adaptado a PRISMA. Este proceso garantizó la trazabilidad y la reproducibilidad del estudio.

### Evaluación de la Calidad

Para minimizar el riesgo de sesgo, se evaluó la calidad metodológica de los estudios incluidos. Los estudios cuantitativos se evaluaron utilizando la lista de verificación Strengthening the Reporting of Observational Studies in Epidemiology (STROBE) y se clasificaron como: A (≥80% de los criterios cumplidos), B (50–79%) o C (<50%, excluidos de la síntesis). Los estudios cualitativos se evaluaron con la herramienta del Programa de Habilidades de Evaluación Crítica (CASP), con una puntuación basada en el cumplimiento de cada criterio: 1 punto (cumplido totalmente), 0,5 puntos (cumplido parcialmente) y 0 puntos (no cumplido). Las puntuaciones oscilaron entre 0 y 10 y se clasificaron como alto rigor (≥9 puntos), rigor moderado (5-8,5) y bajo rigor (<5, excluido de la metasinopsis).

### Registro

Esta revisión se registró prospectivamente en la base de datos PROSPERO el 13 de mayo de 2024, con el número de registro CRD42024541613.

### Criterios de Elegibilidad, Análisis de los Artículos Revisados y Metodología de Triangulación

#### Proceso de Selección y Análisis de los Estudios

Las búsquedas identificaron un total de 2010 registros, que se importaron al software Rayyan para su selección inicial. En la primera etapa, se eliminaron 578 duplicados. Posteriormente, tres revisores examinaron de forma independiente los títulos y resúmenes, y se excluyeron 773 estudios por estar fuera del ámbito de aplicación. Tras este paso, se evaluaron 659 artículos en su versión completa. De ellos, se excluyeron 577 por no cumplir los criterios de elegibilidad, lo que dio como resultado 82 artículos potencialmente relevantes. Tras un análisis detallado, se incluyeron 27 estudios en la metasinopsis final (Figura 1).

**Figura 1:** Estrategia de búsqueda de los estudios incluidos en la investigación.

Basado en: Moher et al.^(8)^. para más información, visite el sitio web de PRISMA^(9)^

#### Criterios de Elegibilidad

Se incluyeron los estudios originales que: (i) abordaban la tuberculosis en la población en situación de calle de América del Sur; (ii) analizaran determinantes sociales, individuales o programáticos; y (iii) informaran sobre resultados relacionados con la mortalidad, la adherencia o el abandono del tratamiento. Se excluyeron los artículos que: (i) no se centraban en la población en situación de calle; (ii) se limitaban a estimaciones descriptivas de prevalencia o riesgo sin análisis de determinantes; (iii) evaluaban únicamente la eficacia de los medicamentos sin tener en cuenta determinantes sociales más amplios; o (iv) eran editoriales, revisiones narrativas o informes de casos.

#### Metasíntesis y Triangulación

El análisis de los 27 estudios siguió el método de metasíntesis, estructurado en torno a tres pilares:

Metateoría: evaluación del marco conceptual subyacente a los estudios, abordando temas como el acceso a los servicios de salud, las condiciones de vida en la calle, la inseguridad alimentaria, el estigma social, la resistencia a los medicamentos, el abandono del tratamiento y la mortalidad^(10,11)^.

Metamétodo: examen de la coherencia de los diseños metodológicos, destacando las fortalezas y limitaciones de las estrategias utilizadas y su capacidad para generar evidencia consistente y relevante^(11,12)^.

Metaanálisis cualitativo: integración temática de los resultados, identificando determinantes, vulnerabilidades y resultados clínicos y epidemiológicos^(11,13)^.

Se aplicó la triangulación metodológica para integrar los resultados cuantitativos y cualitativos. Se revisaron los siguientes aspectos para la extracción de datos: título del estudio, tipo de estudio, factores de vulnerabilidad y principales resultados^(14,15)^.

Se consideró la convergencia cuando dos o más estudios presentaban patrones similares, como la influencia de la pobreza extrema, el consumo de sustancias y el estigma social en el abandono del tratamiento^(14)^. La divergencia reflejaba variaciones regionales, contextuales y metodológicas, como la diferente prevalencia de la coinfección por TB-VIH en los distintos países, lo que ponía de relieve la necesidad de intervenciones personalizadas y adaptadas a los contextos locales específicos^(15)^.

## RESULTADOS

El Cuadro 1 destaca la vulnerabilidad de la población en situación de calle a la tuberculosis (TB) en América del Sur, con tasas de fracaso terapéutico del 38,63%. Silva et al.^(16)^ informaron que en Brasil, 14 059 personas registradas en los Sistemas de Información Sanitaria (*SINAN*) presentaban una tasa de abandono del tratamiento del 35,2% (p < 0,05). En Roraima, Soares et al.^(17)^ identificaron tasas de abandono superiores al 40% (desviación estándar ±5,1), con el mayor impacto en la población inmigrante. Estudios transversales, como el de Gioseffi et al.^(18)^, destacaron una mayor probabilidad de abandono entre los consumidores de alcohol y drogas (odds ratio 2,3; IC del 95%, 1,8–2,9). Además, Ranzani et al.^(19)^ describieron el fracaso del tratamiento en el 57,3% de los casos en la población en situación de calle, con una alta mortalidad asociada a la coinfección por tuberculosis y VIH.

**Cuadro 1:** Resumen de los estudios incluidos.

Autores	Tipo de estudio	Lugar en América del Sur	Población	Variable analizada	Vullnerabilidad programática	Resultados
Gioseffi et al.^(18)^	Transversal	Brasil.	Población en situación de calle en el municipio de Río de Janeiro.	Género, raza/color, VIH, tipo de tuberculosis, consumo de alcohol, tabaco, drogas ilícitas, DOT, distrito de notificación	High population density, socioeconomic barriers, stigma, and lack of social protection.	High treatment dropout rate and greater likelihood of abandonment among alcohol and drug users.
Macedo et al.^(34)^	Descriptiva con análisis de regresión logística.	Brasil.	Personas privadas de libertad (53,6%) (PPL) y población en situación de calle (54,5%) (PSR).	Curación, abandono del tratamiento, muerte relacionada con la tuberculosis, otras causas, tuberculosis multirresistente, cambios en el régimen de tratamiento, nivel educativo, comorbilidades (SIDA, diabetes y enfermedades mentales), alcohol, tabaco y drogas ilícitas.	Pobreza, falta de protección social, barreras económicas y sociales.	La población en situación de calle (PSC) presenta tasas más elevadas de fracaso terapéutico y mortalidad relacionada con la tuberculosis, mientras que el encarcelamiento (PPL) actúa como factor protector contra el fracaso.
Cadorin y Maggi^(35)^	Enfoque cuantitativo transversal.	Brasil.	18 casos reportados en la población en situación de calle (PSC) en Acre.	Incidencia, prevalencia, contactos examinados, tipo de ingreso, cultivo de esputo, VIH y resultados del tratamiento.	Pobreza, falta de programas sociales y falta de protección social.	Hay una alta tasa de curación (50%), tasa de abandono (16.7%) y tasa de mortalidad (11.1%).
Soares et al.^(17)^	Cuantitativo, transversal y descriptivo.	Brasil.	Casos confirmados de tuberculosis en el estado de Roraima entre 2009 y 2019.	Perfil sociodemográfico, incluyendo características clínicas, diagnóstico y seguimiento del caso.	Pobreza, falta de protección social y mayor exposición a condiciones adversas para los migrantes.	Los migrantes que se encuentran en la Población en situación de calle (PSC) presentan tasas de abandono más elevadas y menores índices de éxito en los tratamientos, lo que pone de manifiesto importantes desigualdades.
Rodrigues^(36)^	Descriptivo, cuantitativo.	Brasil.	Población en situación de calle en Brasil en el año 2022.	Género, edad, infecciones por VIH, tabaquismo, alcoholismo, comorbilidad y resultados del tratamiento.	Estigma, pobreza, falta de protección social.	Alta tasa de abandono del tratamiento (181 casos) y presencia de DOT en casos confirmados de sida.
Carneiro et al.^(37)^	Estudio transversal, observacional con enfoque cuantitativo.	Brasil	La población en situación de calle en el municipio de Passos (MG), con 78 entrevistas.	Género, edad, nivel educativo, lugar donde duerme, duración de la situación de población en situación de calle, fuente de ingresos, contactos con personas con tuberculosis, consumo de alcohol, tabaco y drogas ilícitas, comorbilidades y acceso a la higiene.	Pobreza, falta de protección social, nomadismo, estigma y ruptura de los lazos familiares.	No se especifica. Se informa de que la alta prevalencia de factores de riesgo aumenta la vulnerabilidad a la tuberculosis.
Santos et al.^(14)^	Estudio transversal con enfoque cuantitativo.	Brasil	1530 pacientes con tuberculosis en situación de calle registrados en CadÚnico.	Género y raza/color.	Experimentar la situación de calle y la pobreza extrema.	Alta tasa de fracaso del tratamiento, evidenciada por un alto porcentaje de abandono del tratamiento y una tasa de mortalidad significativa, junto con una probabilidad de curación relativamente baja en comparación con la población general.
Ranzani et al.^(38)^	Longitudinal con enfoque cuantitativo.	Brasil	Población con tuberculosis en el estado de São Paulo (2010 a 2015).	Género, edad y raza/color.	Factores sociales, hábitos de salud y comorbilidades.	Una tasa de curación elevada, pero aún con una proporción significativa de intervenciones necesarias para mejorar el seguimiento y reducir las vulnerabilidades sociales y sanitarias que afectan a estos pacientes.
Ranzani et al.^(19)^	Cuantitativo, retrospectivo.	Brasil	Población en situación de calle con tuberculosis pulmonar en el estado de São Paulo (2009–2013).	Género, color de piel, consumo de alcohol y drogas, tipo de tuberculosis, VIH, diabetes y resultados del tratamiento.	No se menciona.	Tasa de fracaso del tratamiento del 57,3%, caracterizada por altas tasas de abandono y mortalidad.
Snyder et al.^(20)^	Cuantitativo, retrospectivo.	Brasil	Personas que viven en grupos familiares o no- familiares, en situación de calle, que informaron haber padecido tuberculosis en 2010.	Género, tipo de tuberculosis, VIH, resultados del tratamiento (curación y mortalidad).	No mencionado.	El uso de la terapia directamente observada (DOT, por sus siglas en inglés) condujo a un aumento de las tasas de curación hasta el 54% y a una reducción de las tasas de abandono del tratamiento hasta el 16% y de las tasas de mortalidad hasta el 6% entre la población que vive en asentamientos informales y la Población en situación de calle.
Rodrigues et al.^(21)^	Cuantitativo, retrospectivo	Brasil	Población en situación de calle con tuberculosis (2015–2020).	Género, edad, raza/color, tabaquismo, alcoholismo, abuso de drogas, VIH, diabetes, tipo de tuberculosis, resultados y tratamiento (curación, mortalidad y abandono)	No mencionado.	Fracaso del tratamiento en el 57.3% de los casos de abandono y mortalidad. Los principales factores predictivos fueron el consumo de drogas y la ausencia de DOT.
Aguiar et al.^(40)^	Cuantitativo, descriptivo, y transversal.	Brasil.	Personas encarceladas y personas de la población en situación de calle con tuberculosis (2015–2017).	Género, edad, raza/color, nivel educativo, VIH, alcoholismo, tabaquismo, tipos de tuberculosis, resultados del tratamiento.	Sistema sanitario deficiente y falta de protección social.	El abandono del tratamiento es el principal resultado en la población en situación de calle (PSC), con un bajo uso de pruebas moleculares y DOT.
Sousa et al.^(45)^	Cualitativo, descriptivo y exploratorio.	Brasil	Personas mayores de 18 años que vivían con VIH/SIDA y estaban coinfectadas con tuberculosis en 2011.	Consumo de alcohol, drogas ilícitas, cumplimiento del tratamiento, dificultades sociales y económicas relacionadas con el estilo de vida de la Población en situación de calle.	No mencionado.	Las barreras sociales y económicas y el estilo de vida que dificultan la adherencia al tratamiento, con una tasa de abandono del 7,6%.
Ranzani et al.^(43)^	Epidemiológico y descriptivo con análisis de tendencias y modelización.	Brasil, Argentina, Chile, Colombia, Ecuador, El Salvador, Perú, Uruguay y Venezuela.	La edad, el género y las personas con VIH estratifican a la población general.	Edad, género, aumento de la incidencia de la tuberculosis, factores económicos y sociales.	Sistema sanitario deficiente y falta de protección social.	Aumento de las tasas de incidencia, mortalidad, abandono y desafíos en el control regional de la tuberculosis en cada país sudamericano.
Maffacciolli et al.^(25)^	Cualitativo, descriptivo, y exploratorio.	Brasil	12 personas de la población en situación de calle (6 hombres y 6 mujeres) con tuberculosis.	Trayectorias de hospitalización, historia de vida, consumo de drogas y dinámica de las relaciones afectivas y sexuales.	Sistema de salud deficiente en la prestación de atención a la población en situación de calle (PSC).	Identificación de vulnerabilidades sociales, económicas y sanitarias asociadas.
Pavinati et al.^(26)^	Descriptivo, ecológico, geoespacial.	Brasil.	Adultos de la Población en situación de calle (PSC) con tuberculosis.	Indicadores epidemiológicos, resultados del tratamiento, disparidades regionales y temporales	Sistema de salud deficiente.	Altas tasas de abandono y mortalidad, desigualdades regionales en el desempeño de los indicadores epidemiológicos
Freitas et al.^(27)^	Cuantitativo y descriptivo.	Brasil	21 841 casos de tuberculosis (21 305 en la población general, 349 en la población reclusa y 187 en la Población en situación de calle).	Prueba molecular rápida (RMT), tiempo entre el diagnóstico y el inicio del tratamiento, tasa de pruebas del VIH, DOT, curación y abandono del tratamiento.	Falta de protección social, datos faltantes o incorrectos, baja utilización del RMT.	El DOT se realizó solo en el 22% de la población en situación de calle (PSC), con altas tasas de abandono y mortalidad.
Zuim y Trajman^(28)^	Cualitative y interpretativo.	Brasil.	19 pacientes adultos de la Población en situación de calle (PSC) con tuberculosis.	Género, edad, raza/color, nutrición, consumo de alcohol, abuso de drogas, VIH, fragmentación de la atención y estigma	Sistema sanitario deficiente y falta de protección social.	Estigma, abandono del tratamiento, mortalidad relacionada con la tuberculosis y deficiencias en la atención continua.
Lima et al.^(29)^	Descriptivo, exploratorio, y Cuantitativo.	Brasil.	70 personas en situación de calle.	Género, grupo de edad, raza/color, nivel educativo, tabaquismo, alcoholismo, uso de servicios de salud pública, pobreza, resultados del frotis de esputo	Sistema sanitario deficiente y falta de protección social.	Alta tasa de abandono en la prevalencia del abandono y vulnerabilidad social a la tuberculosis.
Fica et al.^(30)^	Descriptivo, transversal.	Chile.	Población general y grupos especiales (población en situación de calle).	Género, edad, VIH/SIDA, consumo de alcohol y drogas, pobreza, indicadores del programa de control de la tuberculosis (PROCET).	Sistema sanitario deficiente y falta de modernización en el diagnóstico.	Aumento de la incidencia de tuberculosis, alta letalidad (>3%), pérdida de seguimiento (>5%), baja tasa de curación (<90%) y aumento de la tuberculosis entre la Población en situación de calle.
Sales et al.^(42)^	Ecológico y descriptivo.	Brasil.	Se analizaron 577 sectores censales (588 casos de tuberculosis entre 2009 y 2011).	Género, edad, educación, ingresos, acceso a servicios básicos de saneamiento, subregistro de casos y distribución espacial.	Sistema sanitario deficiente, baja vigilancia epidemiológica y alto nivel de subnotificación.	Identificación de subnotificación en el 93% de los sectores sin casos notificados y mayor prevalencia de tuberculosis en zonas de bajos ingresos y bajo nivel educativo.
Hino et al.^(32)^	Historia oral cualitativa y temática.	Brasil.	24 personas de la población en situación de calle (PSC) en tratamiento contra la tuberculosis.	Experiencia de vida, adherencia al tratamiento, percepción de los servicios de salud.	Sistema sanitario deficiente y falta de protección social.	Obstáculos para el tratamiento asociados con la vida en la calle, perspectivas de curación como motivación para seguir el tratamiento.
Silva et al.^(33)^	Ecológico.	Brasil.	2966 casos de TB (544 casos de abandono).	Grupo de edad, género, raza/color, educación, VIH, tipo de tuberculosis y afecciones de salud asociadas (alcohol, drogas).	Sistema de salud deficiente, falta de protección social, obstáculos para acceder al tratamiento.	18.7% de abandono del tratamiento, con un aumento progresivo en años específicos.
Rodrigues y Tauil^(41)^	Epidemiológico y ecológico.	Brasil.	Nuevos casos de tuberculosis entre 2006 y 2015.	Género, edad, VIH, tabaquismo, alcoholismo, diabetes, drogas ilícitas, presentación clínica.	Sistema de salud deficiente.	Reducción de la incidencia de la tuberculosis, disminución de la tasa de curación, aumento del abandono con el tiempo.
Pinheiro et al.^(31)^	Observacional y descriptivo.	Brasil.	127 536 personas en situación de calle (PSH) registradas en CadÚnico y 14 059 con tuberculosis.	Género, grupo de edad, raza y color, nivel educativo, beneficiarios del gobierno.	Déficit en la asistencia sanitaria y falta de protección social.	Déficit en la asistencia sanitaria y falta de protección social.
Pinheiro et al.^(22)^	Revisión integrativa de la literatura.	Brasil.	Población en situación de calle (personas sin hogar) con tuberculosis.	Trayectorias asistenciales; prácticas de cuidado; adherencia al tratamiento; papel de los Equipos de Consultorio en la Calle (CnaR); determinantes sociales (consumo de sustancias, inestabilidad habitacional, inseguridad alimentaria).	Alta vulnerabilidad programática y social: barreras de acceso a los servicios de salud, discontinuidad del cuidado, fragmentación de los servicios, estigma institucional, invisibilidad social, débil articulación intersectorial.	Baja adherencia asociada a condiciones sociales adversas; abandono del tratamiento influenciado por el consumo de sustancias y condiciones de vida inestables; mejora de la adherencia con enfoques integrados y adaptados; el CnaR como dispositivo clave para la construcción de vínculos y la garantía del cuidado integral.
Silva et al.^(16)^	Estudio descriptivo y transversal.	Brasil.	Población en situación de calle (PSC) en CadÚnico (2014 a 2019).	Género, grupo de edad, raza/color, nivel educativo, ingresos, falta de protección social y desigualdad en el acceso.	Déficit en la asistencia sanitaria y falta de protección social.	Alta tasa de abandono y baja cobertura del tratamiento.

Fuente: Autores, 2024.

La terapia directamente observada aumenta las tasas de curación del 54% al 67% (p < 0,05) en la población en situación de calle, según Snyder et al.^(20)^ (Tabla 1). Triangulación de datos de los estudios analizados, en materiales resumidos). El análisis de la Tabla 1 destaca los aspectos convergentes y divergentes relacionados con el perfil socioeconómico, los resultados del tratamiento, las vulnerabilidades programáticas, la distribución geográfica y los ingresos/beneficios sociales en la población en situación de calle con tuberculosis de América del Sur.

En el perfil socioeconómico, todos los estudios coincidieron en el predominio de hombres de entre 20 y 49 años, con bajos niveles educativos y una alta proporción de personas negras y mestizas, considerando las vulnerabilidades raciales y educativas como factores de riesgo. La convergencia media en este aspecto fue del 74%. En cuanto a los resultados del tratamiento, se describieron altas tasas de abandono en estudios como el de Rodrigues et al.^(21)^, con un 36,02% de abandonos y un 14,37% de mortalidad, mientras que Pinheiro et al.^(22, 31)^ informaron de que la tasa de curación disminuyó del 39,6% al 34,5% entre 2014 y 2018 en la región. La convergencia media fue del 68%. Sin embargo, pocos estudios (n = 09) exploraron estrategias para revertir el aumento de los abandonos, como las intervenciones basadas en el DOT. En cuanto a la vulnerabilidad programática, el 65% de los estudios señalaron la insuficiencia de la protección social como factor determinante, con una baja cobertura de programas sociales como *Bolsa Família* (4% en Silva et al.^(16)^).

Sin embargo, no se describió ningún consenso en cuanto a la implementación de políticas intersectoriales para mitigar los impactos de la pobreza extrema en la adherencia al tratamiento. La convergencia promedio fue del 67%. La distribución geográfica destacó una concentración en las zonas urbanas de América del Sur, con algunas regiones como el norte y el noreste que siguen estando infrarrepresentadas. Solo el 12% de los estudios describieron la movilidad geográfica de la población en situación de calle como una barrera para la atención de salud, haciendo hincapié en la necesidad de políticas de salud adaptadas a las zonas remotas y fronterizas.

Por último, en lo que respecta a los ingresos y las prestaciones sociales, la pobreza extrema predominaba en el 90% de las poblaciones en situación de calle analizadas en los estudios, pero los impactos directos de los programas sociales en la mejora de los resultados siguen sin evaluarse adecuadamente. La convergencia media fue del 72%.

El análisis de los estudios revela que el 12% abordó el papel de la inestabilidad temporal de la vivienda, como el uso de refugios públicos, como factor intermedio en la adherencia al tratamiento y el seguimiento clínico. Además, menos del 10% abordó la influencia de los factores culturales en la aceptación de las intervenciones terapéuticas entre los subgrupos de la población en situación de calle, lo que plantea la necesidad de enfoques que aborden la heterogeneidad de esta población. Este análisis subraya la importancia de integrar datos longitudinales y contextuales para comprender mejor las dinámicas sociales que afectan a los resultados de salud de la población en situación de calle en América del Sur.

En la Figura 2 se destaca la relación entre la distribución geográfica de las poblaciones en situación de calle y las divergencias en las vulnerabilidades programáticas para el tratamiento de la tuberculosis, particularmente en el contexto del Tratamiento Directamente Observado (Directly Observed Treatment (DOT)). Aunque el DOT se utiliza como una estrategia eficaz en los estudios analizados, la convergencia incompleta (75%) describe obstáculos específicos para su implementación, como la dificultad de mantener la continuidad de la atención entre poblaciones que se desplazan con frecuencia, como las personas en situación de calle.

**Figura 2:** Convergencia y divergencia en la tuberculosis entre la población en situación de calle en América del Sur, 2024.

**Fuente:** Autores, 2024.

Otro aspecto relevante es el impacto de los programas sociales transitorios, como la ayuda de emergencia, en la adherencia al tratamiento de la población en situación de calle. La alta convergencia en las prestaciones sociales (80%) contrasta con la ausencia de estudios que evalúen cómo estas políticas temporales afectan a la continuidad del tratamiento a largo plazo, especialmente en zonas urbanas densamente pobladas. Además, la relación entre los factores climáticos y la adherencia al DOT sigue siendo una laguna descuidada en la literatura. Estudios como los de Soares et al.^(17)^ sugieren retos regionales, pero no relacionan directamente la estacionalidad con los resultados terapéuticos. En el análisis de las regiones fronterizas, como Roraima, se destacan los retos que plantean las intensas lluvias, que dificultan el acceso a los servicios de salud de la población en situación de calle, según informan Soares et al.^(17)^

En la Figura 3, destaca la falta de protección social (85%) como el principal determinante identificado en los estudios analizados, seguida del abandono del tratamiento (80%) y la pobreza extrema (75%). También se destacan la concentración urbana (70%) y el consumo de drogas ilícitas (65%). Otras vulnerabilidades, como las deficiencias programáticas (60%), el estigma (55%), los bajos niveles de educación (50%) y las comorbilidades de la tuberculosis y el VIH (45%), reflejan barreras sociales y estructurales persistentes. En Figura 2, las vulnerabilidades sociales y programáticas muestran una media de 1,75 (IC del 95%: 1,50–2,00), lo que indica el fuerte impacto de este determinante en los resultados de la tuberculosis.

**Figura 3:** Metaanálisis de las vulnerabilidades en los estudios sobre la tuberculosis en la población en situación de calle.

**Fuente:** Autores, 2024.

En la Figura 2 y Figura 3, las vulnerabilidades geográficas muestran un efecto moderado, con un promedio de 1,10 (IC del 95%: 1,05–1,20), lo que caracteriza las desigualdades regionales como factores influyentes en el tratamiento de la tuberculosis en la región. Por otro lado, los beneficios sociales tienen un impacto menor, con un promedio de 1,05 (IC del 95%: 1,00–1,15), lo que sugiere una cobertura y una implementación insuficientes. Los resultados del tratamiento, como el abandono y la mortalidad, mostraron un tamaño del efecto de 1,25 (IC del 95%: 1,15–1,35), lo que refuerza la influencia de los determinantes sociales y estructurales en las respuestas al tratamiento de la tuberculosis.

En Figura 2 y Figura 3, se describe el riesgo relativo (RR) asociado a las vulnerabilidades sociales en la población en situación de calle con tuberculosis. La coinfección por VIH presenta el riesgo relativo más alto (RR = 3,0, IC del 95%: 2,5–3,5), seguida del consumo de drogas ilícitas (RR = 2,5, IC del 95%: 2,0–3,0) y el alcoholismo (RR = 2,0, IC del 95%: 1,7–2,5). En el gráfico 2, la inestabilidad de la vivienda y la pobreza extrema también aumentan el riesgo, con RR de 1,8 (IC del 95%: 1,4–2,2) y 1,7 (IC del 95%: 1,5–2,0), respectivamente. El acceso limitado al DOT y la cobertura insuficiente de las prestaciones sociales muestran RR moderados, en torno a 1,5 (IC del 95%: 1,2–1,8), lo que pone de relieve las áreas prioritarias para las intervenciones.

## DISCUSIÓN

Los resultados obtenidos del análisis de los 27 estudios incluidos destacan diferentes determinantes de la vulnerabilidad asociada a la TB en las PSC en América del Sur. Estos hallazgos revelan la intersección de vulnerabilidades individuales, sociales y programáticas, haciendo hincapié en los factores que perpetúan las desigualdades en el acceso al diagnóstico, el tratamiento y los resultados favorables^(15,23,24,25,26,27)^. En el contexto programático, la limitada implementación de políticas dirigidas a la población de alto riesgo, como los Consultorios en la Calle, con solo el 60% de los equipos previstos en activo en 2019, indica la necesidad de una mayor priorización gubernamental en Brasil. Las reformas de los sistemas de información y el fortalecimiento de las redes de apoyo se perfilan como medidas esenciales para mejorar la atención, especialmente en las regiones con una mayor concentración de vulnerabilidades^(15,23,24,25,26,27)^.

En cuanto a las vulnerabilidades individuales, los estudios analizados destacan la influencia del consumo de drogas ilícitas, alcohol y otras sustancias en la adherencia al tratamiento de la tuberculosis. Esta asociación se relacionó con tasas de abandono del tratamiento de hasta el 33% en algunas regiones de Brasil y otros países sudamericanos, con un riesgo relativo 2,5 veces mayor entre los consumidores de sustancias, un valor siete veces superior al umbral aceptable definido por la OMS^(16,18,28,29,30,31,32,33)^.

La movilidad constante, el nomadismo y la ruptura de los lazos familiares aumentan las barreras para acceder a los servicios de salud y comprometen la continuidad de la atención, mientras que las comorbilidades, como los trastornos mentales y la coinfección por TB-VIH, exacerban los retos a los que se enfrenta la población vulnerable^(16,18,34,35)^. La coinfección por TB-VIH, reportada en el 45% de los estudios, refleja la complejidad de estos determinantes, ya que se asocia con un aumento de la mortalidad y la intensificación del estigma social^(16,18,34,35)^.

El perfil predominante identificado, con una edad media de 49,8 años y una mayor prevalencia entre los hombres adultos jóvenes, también sugiere una mayor vulnerabilidad asociada a la movilidad de esta población^(16,18,34,35)^. En términos de vulnerabilidades sociales, las condiciones de vida precarias y la exclusión sistémica refuerzan las desigualdades estructurales. Los estudios mostraron que el 63,6% de las personas pertenecían a grupos negros o mestizos, lo que pone de relieve cómo las disparidades raciales y económicas refuerzan la marginación, especialmente en Brasil^(16,18,32,33)^.

Los antecedentes de encarcelamiento, mencionados de forma recurrente en las publicaciones, indican ciclos prolongados de exclusión social, en los que las personas recién liberadas se enfrentan al estigma, la falta de oportunidades económicas y el apoyo institucional insuficiente, lo que a menudo culmina en la falta de hogar^(16,18,34,35)^. La pobreza extrema, identificada en el 75% de las publicaciones, dificulta aún más el acceso a los servicios básicos de salud y a los programas de asistencia social, mientras que la baja cobertura de las prestaciones sociales como la Bolsa Família, especialmente en las regiones del norte y noreste de Brasil, constituye una barrera crítica para el control de la tuberculosis entre la PSC^(34,35)^.

También se evidenció ampliamente la vulnerabilidad de los programas. Los estudios indicaron que el 57% de los casos de tuberculosis en las PSC tuvieron resultados desfavorables, y que el consumo de drogas (OR = 1,54; IC del 95%: 1,31–1,80) y la ausencia de tratamiento supervisado (OR = 0,52; IC del 95%: 0,45–0,60) aumentaban significativamente el riesgo de abandono del tratamiento y mortalidad^(16,18,34,35)^. En las regiones fronterizas, como Roraima, la limitada infraestructura sanitaria, junto con las barreras climáticas y logísticas, comprometían el diagnóstico y el tratamiento oportunos^(16,18,34,35)^. La cobertura limitada de la prueba molecular rápida (Rapid Molecular Test – RMT-TB), especialmente en grandes centros urbanos como São Paulo, Belo Horizonte y Río de Janeiro, ilustra fallos críticos en la gestión de casos^(17,36,37)^.

Los análisis también indicaron que las políticas sanitarias, a menudo desconectadas de la realidad local, no incorporan plenamente los determinantes sociales, económicos y ambientales que influyen en los resultados de la PSC^(17,36,37)^. Esta limitación se ve agravada por la baja cobertura de la DOT, que está directamente relacionada con el aumento del abandono y la mortalidad^(17,20,36,37,38)^.

A pesar de los avances, persisten importantes lagunas. La inestabilidad temporal de la vivienda, como las estancias en refugios, se ha explorado poco como factor intermedio para la adherencia al tratamiento. Además, los factores climáticos y ambientales siguen estando muy descuidados, a pesar de su posible influencia en la incidencia y el manejo de la tuberculosis, como se observa en las regiones fronterizas afectadas por fuertes lluvias ^(19,21,39,40,41)^.

La diversidad cultural y regional de América del Sur también exige análisis específicos del contexto que reconozcan las particularidades locales. En este escenario, las acciones intersectoriales que integran las políticas de salud con los programas sociales, como la ampliación de las prestaciones de protección social, son esenciales para mitigar la pobreza extrema y reducir las desigualdades^(19,21,39,40,41)^. Estas iniciativas deben alinearse con la Agenda 2030 y los ODS, garantizando la salud como un derecho humano básico y promoviendo la equidad mediante la integración de la protección social, las tecnologías sanitarias y las intervenciones adaptadas a los contextos territoriales y climáticos^(22,42,43,44,45,46,47)^.

## CONCLUSIÓN

El análisis de los 27 estudios incluidos pone de relieve las múltiples vulnerabilidades a las que se enfrentan la PSC en América del Sur en el contexto de la tuberculosis. El perfil predominante se caracterizó por hombres jóvenes, con una mayor proporción de personas negras y mestizas, bajos niveles educativos y que vivían en condiciones de pobreza extrema, lo que refleja las persistentes desigualdades estructurales que repercuten directamente en los resultados clínicos y epidemiológicos.

Las elevadas tasas medias de abandono del tratamiento (36%) y mortalidad (14%) están fuertemente asociadas a barreras sociales y programáticas, como la cobertura insuficiente de las prestaciones sociales (85%) y la aplicación limitada de programas de protección, como *Bolsa Família*. El análisis geográfico también reveló la infrarrepresentación de las regiones remotas y fronterizas, donde la movilidad de la población y las condiciones climáticas adversas aumentan las dificultades para acceder oportunamente a los servicios de salud.

Aunque el DOT se reconoce como una estrategia eficaz en Brasil, su cobertura sigue estando marcada por las desigualdades regionales y las limitaciones estructurales, lo que compromete la adherencia y el éxito del tratamiento. Aspectos como la inestabilidad temporal de la vivienda y la influencia de los factores climáticos en los resultados del tratamiento apenas se han explorado, lo que indica la necesidad de realizar análisis longitudinales que integren los determinantes sociales, ambientales y programáticos. A la luz de estos hallazgos, se requiere urgentemente la formulación y expansión de políticas públicas intersectoriales, basadas en el principio de equidad y adaptadas a las necesidades específicas de la población en situación de calle.

La incorporación de medidas para fortalecer la protección social, ampliar la cobertura del DOT, integrar tecnologías de diagnóstico y considerar los factores territoriales y climáticos es esencial para abordar las desigualdades regionales y promover mejoras sostenibles en los indicadores de salud de la población en situación de calle en América del Sur.

## Data Availability

Todo el conjunto de datos que apoya los resultados de este estudio fue publicado en el propio artículo.
